# Role of TREM-1 in pulmonary tuberculosis patients- analysis of serum soluble TREM-1 levels

**DOI:** 10.1038/s41598-018-26478-2

**Published:** 2018-05-29

**Authors:** Jia-Yih Feng, Wei-Juin Su, Sheng-Wei Pan, Yi-Chen Yeh, Yung-Yang Lin, Nien-Jung Chen

**Affiliations:** 1 Department of Chest Medicine, Taipei Veterans General Hospital, Taipei, Taiwan; 2 School of Medicine, National Yang-Ming University, Taipei, Taiwan; 3 Institute of Clinical Medicine, School of Medicine, National Yang-Ming University, Taipei, Taiwan; 4 Institute of Public Health, National Yang-Ming University, Taipei, Taiwan; 5 Department of Pathology and Laboratory Medicine, Taipei Veterans General Hospital, Taipei, Taiwan; 6 Division of Cerebrovascular Diseases, Neurological Institute, Taipei Veterans General Hospital, Taipei, Taiwan; 7 Institute of Brain Science, National Yang-Ming University, Taipei, Taiwan; 8 Institute of Microbiology and Immunology, School of Life Sciences, National Yang-Ming University, Taipei, Taiwan

## Abstract

Triggering receptor expressed on myeloid cells 1 (TREM-1) amplifies inflammatory responses and is upregulated during sepsis and pulmonary infection. The association between serum soluble TREM-1 (sTREM-1) level and pulmonary tuberculosis (PTB) disease deserves investigation. In the present study, patients with PTB, latent TB infection (LTBI), and non-TB, non-LTBI subjects were prospectively enrolled and serum levels of sTREM-1, sTREM-2, and C-reactive protein (CRP) were measured. We correlated serum biomarkers and clinical presentations and treatment outcomes of PTB cases. We also utilized immunohistochemistry (IHC) to visualize TREM-1-expressing cells in lung tissues from PTB patients. A total of 86 PTB, 41 LTBI, and 20 non-TB, non-LTBI subjects were enrolled. Serum levels of sTREM-1 and CRP significantly increased in PTB patients; these higher serum levels were correlated with more advanced involvement in chest films and higher bacteria burden in sputum. In multivariate analysis, serum levels of sTREM-1 >260 pg/mL and CRP >2.6 mg/L were independent predictors for on-treatment mortality. Abundant TREM-1-expressing macrophages were identified in lung tissues from PTB samples. In conclusion, serum levels of sTREM-1 correlated with disease severity and treatment outcomes in PTB patients.

## Introduction

Despite effective anti-tuberculosis (TB) therapies, TB remains an infectious disease with high morbidity and mortality. Both innate and adaptive immunity are involved in host defense mechanisms against *Mycobacterium tuberculosis* (MTB) in human lungs. The major innate cells in humans include macrophages, neutrophils, dendritic cells, and natural killer cells. Macrophages play a pivotal role among the innate cells, and alveolar macrophages are the first line of defense against MTB. Through a number of receptors on the cell surface, MTB is recognized and ingested by alveolar macrophages, which in turn activate downstream signaling and induce the production of inflammatory cytokines. Although innate immunity is activated early to limit disease dissemination, recent studies demonstrated that myeloid cells also facilitate the replication of MTB^1,2^.

The triggering receptor expressed on myeloid cells (TREM) family is a group of cell surface receptors expressed on myeloid cells, including monocytes/macrophages and neutrophils^3,4^. After associating with the adaptor DNAX activation protein (DAP)-12, TREM family proteins trigger a signaling pathway that modulates infection-related inflammatory responses^4,5^. Among the TREM family, TREM-1 has the synergistic ability to amplify the signaling of the Toll-like receptors (TLRs) TLR4 or TLR2, which can recognize components of a variety of microorganisms including bacteria, fungi, and viruses^6,7^. In mouse models, engagement of TREM-1 on the cell surface enhances inflammatory reactions, such as cytokine/chemokine production, neutrophil degranulation, and macrophage phagocytosis^8,9^. Unlike TREM-1, the role of TREM-2 in inflammation is still unclear and is likely to be anti-inflammatory. Activation of the TREM-2 pathway can inhibit the production of pro-inflammatory cytokines and promote phagocytosis in macrophages^10,11^. Recent genome-wide studies showed that heterozygous missense mutations in TREM-2 increase the risk of Alzheimer’s disease^12,13^.

Several clinical studies have suggested a specific role of TREM-1 in pneumonia and sepsis. Levels of the soluble form of TREM-1 (sTREM-1) in serum and bronchoalveolar lavage fluid can be used to diagnose bacterial lung infections in intensive care unit (ICU) patients^14^. Increased plasma sTREM-1 is also reported to be a useful marker for evaluating disease severity and outcomes of patients with sepsis^15^. In contrast to bacterial pneumonia and sepsis, information regarding the role of TREM-1 in pulmonary TB is relatively scarce. Considering the potential synergistic effects of TREM-1 in inflammatory reactions mediated by TLR4 and TLR2, which can recognize MTB components, we hypothesize that TREM-1 is also involved in immune responses against MTB. In this study, we measured the serum levels of the soluble forms of TREM-1 and TREM-2 (sTREM-2) in patients with pulmonary TB before initiating anti-TB treatment. The aim of the study was to evaluate the role of sTREMs in clinical presentations and treatment outcomes of pulmonary TB patients. We also used immunohistochemistry (IHC) staining to investigate the presence of TREM-1-expressing cells in lung tissues from patients with pulmonary TB.

## Results

### Patient characteristics

**Figure 1:**
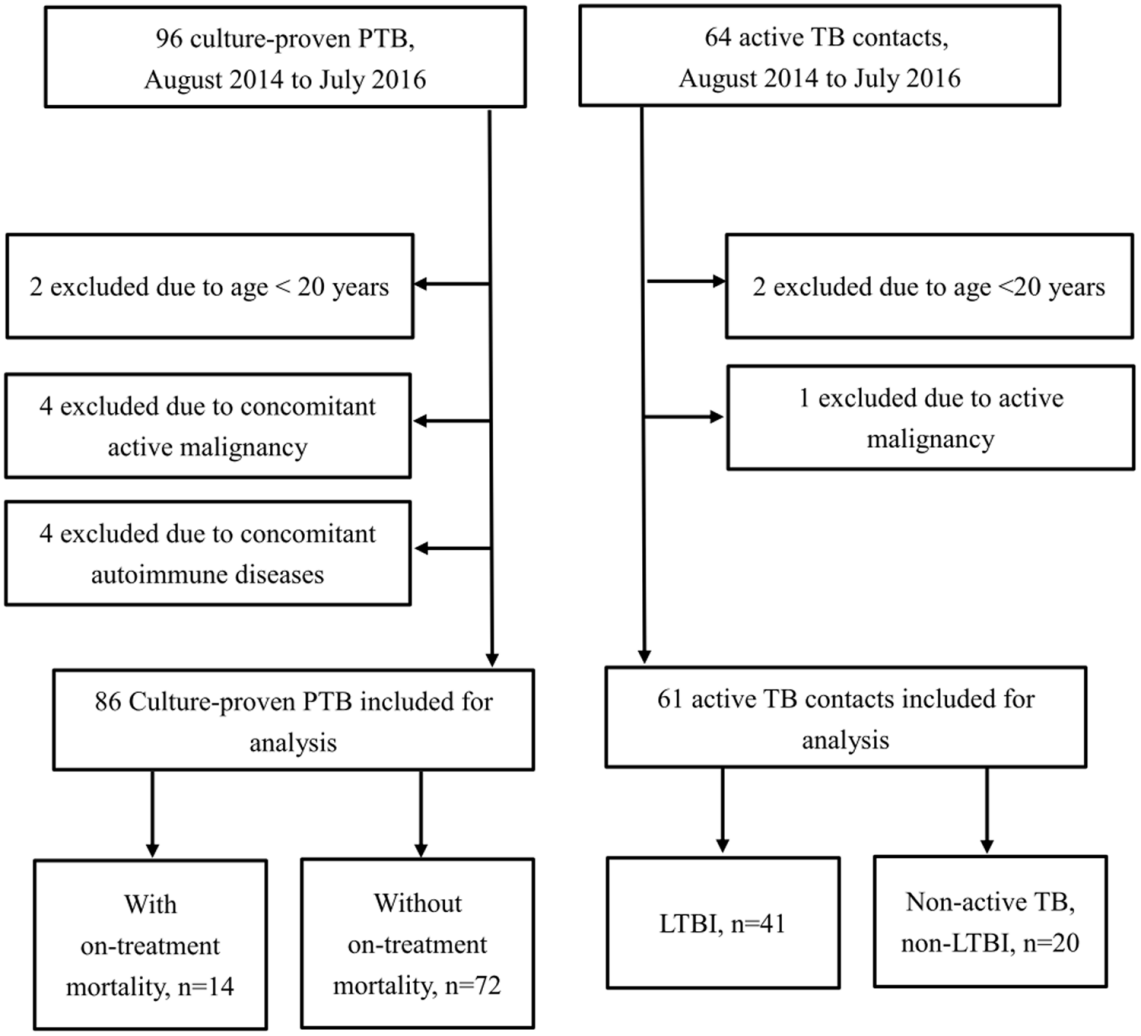
The study profile showing the number of cases and reasons for exclusion.

During the study period, a total of 147 subjects, including 86 active pulmonary TB patients, 41 LTBI patients, and 20 non-TB, non-LTBI subjects, were prospectively enrolled (Fig. 1). The demographic characteristics of the enrolled patients are shown in Table 1. The mean age was 65 ± 18.7 years and 72% were men. Nearly half of the pulmonary TB patients were smokers and 9.3% of them had received TB treatment before. Compared to LTBI cases and non-TB, non-LTBI subjects, pulmonary TB patients were older; more likely to be male, have a smoking habit, and have diabetes mellitus; and were less likely to have Bacillus Calmette-Guérin (BCG) vaccination.

**Table 1:** Demographic characteristics and serum sTREM-1, sTREM-2, and CRP levels in patients with PTB, LTBI, and non-TB, non-LTBI subjects^a^.

	PTB, n = 86	LTBI, n = 41	Non-TB, non-LTBI, n = 20
Mean age (SD)	65.0 (18.7)	47.3 (17.8)***	42.1 (25.1)**
Male sex	62 (72.1%)	19 (46.3%)**	8 (40.0%)**
BMI (SD)	21.7 (4.0)	22.6 (3.4)	20.6 (3.7)
Smoking history	42 (48.8%)	8 (19.5%)**	4 (20.0%)*
BCG vaccination	61 (70.9%)	39 (95.1%)**	18 (80.0%)
Prior TB treatment history	8 (9.3%)	1 (2.4%)	0
Diabetes	17 (19.8%)	5 (12.2%)	0*
Renal insufficiency	4 (5.1%)	0	1 (5%)
COPD	3 (3.5%)	0	0
Malignancy	9 (10.5%)	1 (2.4%)	1 (5%)
Post gastrectomy	2 (2.3%)	0	0
Mean serum biomarker levels			
sTREM1 (mean ± SD)	243.7 ± 212.3	105.8 ± 65.3***	80.1 ± 93.0***
sTREM2 (mean ± SD)	82.8 ± 89.9	84.8 ± 85.5	92.4 ± 114.6
CRP (mean ± SD)	2.93 ± 4.53	0.15 ± 0.22***	0.07 ± 0.07***

^a^Data are presented as mean ± SD or n(%) unless otherwise stated.

SD, standard deviation; BMI, body mass index; BCG, Bacillus Calmette–Guérin; PTB, pulmonary tuberculosis; TB, tuberculosis; LTBI, latent TB infection; COPD, chronic obstructive pulmonary disorder; sTREM, soluble triggering receptor expressed on myeloid cells; CRP, C-reactive protein

* < 0.05, ** < 0.01, *** < 0.001 (compared to PTB patients).

### Serum biomarker levels

**Figure 2:**
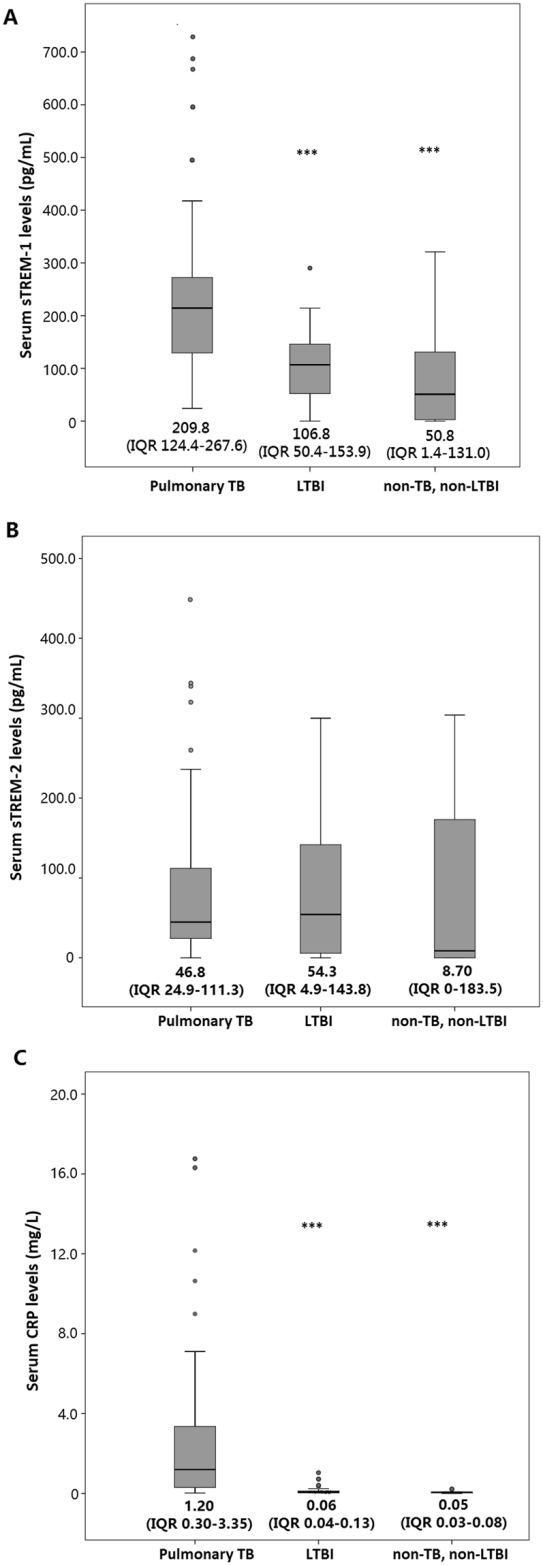
Distribution of serum levels of sTREM-1, sTREM-2, and CRP in patients with pulmonary TB, LTBI, and non-TB, non-LTBI subjects. Data are represented as individual measurements with medians. TB, tuberculosis; sTREM, soluble triggering receptor expressed on myeloid cells; CRP, C-reactive protein.

The distribution and comparison of serum biomarker levels between different groups of patients are shown in Fig. 2. Pulmonary TB patients had significantly higher serum sTREM-1 levels than LTBI patients (p < 0.001) and non-TB, non-LTBI subjects (p < 0.001), although a marked overlap between groups was noted. Patients with pulmonary TB also exhibited significantly higher serum CRP levels than LTBI patients (p < 0.001) and non-TB, non-LTBI subjects (p < 0.001). The serum levels of sTREM-2 were comparable among the three patient groups.

### Serum sTREM-1 and CRP levels correlate with clinical presentations

The association between serum biomarker levels and clinical, radiological, and microbiological presentations in pulmonary TB patients is shown in Table 2. We categorized the patients into subgroups of high and low serum biomarker levels. Patients with higher serum sTREM-1 levels (above the median value, 209.8 pg/ml) were more likely to have poor appetite (p = 0.048), bilateral lung field involvement in chest films (p = 0.009), and positive AFB smears in sputum (p = 0.027). Patients with high serum CRP levels (above the median value, 1.20 mg/L) were more likely to exhibit bilateral lung field involvement in chest films (p = 0.009), and have positive AFB smears in sputum (p = 0.002). On the contrary, patients with high and low serum sTREM-2 levels (above or below median value, 46.8 pg/ml) exhibited similar clinical, radiological, and microbiological presentations.

**Table 2:** Clinical and radiological presentations of pulmonary TB patients with higher and lower serum biomarker levels^a,b^.

	sTREM-1		P value	sTREM-2		P value	CRP		P value
	Lower level	Higher level		Lower level	Higher level		Lower level	Higher level	
Clinical presentations									
Cough	28 (65.1%)	27 (62.8%)	0.822	27 (62.8%)	28 (65.1%)	0.822	29 (67.4%)	26 (60.5%)	0.500
Hemoptysis	4 (9.3%)	1 (2.3%)	0.167	3 (7.0%)	2 (4.7%)	0.645	1 (2.3%)	4 (9.3%)	0.360
Fever	2 (4.7%)	4 (9.3%)	0.397	5 (11.6%)	1 (2.3%)	0.202	2 (4.7%)	4 (9.3%)	0.676
Poor appetite	7 (16.3%)	15 (34.9%)	0.048	9 (20.9%)	13 (30.2%)	0.323	11 (25.6%)	11 (25.6%)	1.000
Weight loss	7 (16.3%)	11 (25.6%)	0.289	10 (23.3%)	8 (18.6%)	0.596	9 (20.9%)	9 (20.9%)	1.000
Radiological presentations									
Cavity	11 (25.6%)	16 (37.2%)	0.245	11 (25.6%)	16 (37.2%)	0.245	10 (23.3%)	17 (39.5%)	0.104
Bilateral	13 (30.2%)	25 (58.1%)	0.009	15 (34.9%)	23 (53.5%)	0.082	13 (30.2%)	25 (58.1%)	0.009
Sputum smear									
Negative	22 (51.2%)	12 (27.9%)	0.027	21 (48.8%)	13 (30.2%)	0.078	24 (55.8%)	10 (23.3%)	0.002
Positive	21 (48.8%)	31 (72.1%)		22 (51.2%)	30 (69.8%)		19 (44.2%)	33 (76.7%)	
1+~2+	13 (30.2%)	13 (30.2%)		9 (20.9%)	17 (39.5%)		12 (27.9%)	14 (32.6%)	
3+~4+	8 (18.6%)	18 (41.9%)		13 (30.2%)	13 (30.2%)		7 (16.3%)	19 (44.2%)	

^a^Data are presented as mean ± SD or n(%) unless otherwise stated.

^b^Higher and lower serum biomarkers levels are defined as levels above or below the median, respectively.

TB, tuberculosis; sTREM, soluble triggering receptor expressed on myeloid cells; CRP, C-reactive protein.

### Association between serum biomarkers levels and treatment outcomes

**Figure 3:**
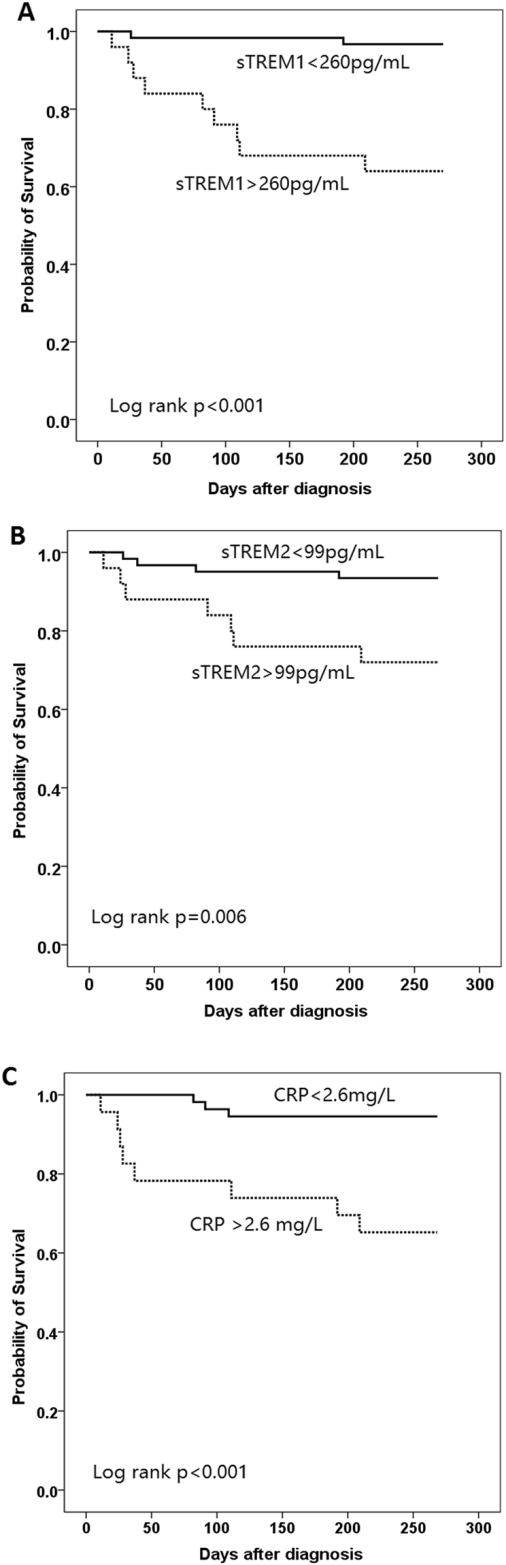
Kaplan-Meier survival curves of patient with pulmonary TB stratified by pre-treatment serum levels of (**A**) sTREM-1, (**B**) sTREM-2, (**C**) CRP. TB, tuberculosis; sTREM, soluble triggering receptor expressed on myeloid cells; CRP, C-reactive protein.

To investigate the association between serum biomarker levels and treatment outcomes in pulmonary TB patients, we compared the 2-month smear conversion rate, 2-month culture conversion rate, and on-treatment mortality rate between patients with high and low serum biomarker levels. As shown in Table 3, pulmonary TB patients with higher sTREM-1 levels had significantly higher on-treatment mortality rate (27.9% vs. 4.7%, p = 0.003) and similar smear and culture conversion rates at 2-months compared to those with low sTREM-1 levels. Patients with pulmonary TB exhibiting higher sTREM-2 levels had significantly higher on-treatment mortality rate (25.6% vs. 7%, p = 0.019) and a trend toward lower smear conversion rate at 2 months (59% vs. 80%, p = 0.051). A trend toward higher on-treatment mortality rate was also noted in pulmonary TB cases with high serum CRP levels, but statistical significance was not reached (23.3% vs. 9.3%, p = 0.080). To determine the best cutoff values for the biomarkers, ROC curves were plotted (data not shown). The calculated best cutoff values to predict on-treatment mortality were 260 pg/mL for sTREM-1, 99 pg/mL for sTREM-2, and 2.6 mg/L for CRP. Kaplan-Meier survival curves categorized by the best cutoff values of each serum biomarkers levels are shown in Fig. 3. Patients with higher levels of sTREM-1, sTREM-2, and CRP exhibited a higher mortality rate (log rank p < 0.001 in sTREM-1, log rank p = 0.006 in sTREM-2, log rank p < 0.001 in CRP). The curves separated early after initiating anti-TB treatment.

**Table 3:** Treatment responses between pulmonary TB patients with higher and lower serum biomarker levels^a,b^.

	sTREM-1		P value	sTREM-2		P value	CRP		P value
	Lower level	Higher level		Lower level	Higher level		Lower level	Higher level	
2 M smear non-conversion, n = 75	29 (74.4%)	22 (62.9%)	0.286	28 (80.0%)	23 (59.0%)	0.051	26 (66.7%)	25 (71.4%)	0.659
2 M culture non-conversion, N = 75	32 (82.1%)	27 (77.1%)	0.600	30 (85.7%)	29 (74.4%)	0.225	32 (82.1%)	27 (77.1%)	0.600
On-treatment mortality	2 (4.7%)	12 (27.9%)	0.003	3 (7.0%)	11 (25.6%)	0.019	4 (9.3%)	10 (23.3%)	0.080

^a^Data are presented as mean ± SD or n(%) unless otherwise stated.

^b^Higher and lower serum biomarkers levels are defined as levels above or below the median, respectively.

TB, tuberculosis; sTREM, soluble triggering receptor expressed on myeloid cells; CRP, C-reactive protein.

Cox regression analyses including clinical characteristics and serum biomarker levels associated with on-treatment mortality in pulmonary TB patients are shown in Table 4. Clinical factors associated with on-treatment mortality in univariate analysis included older age, lower BMI, higher sTREM-1 levels, higher sTREM-2 levels, and higher CRP levels. In multivariate analysis, independent factors associated with higher mortality included older age (HR 1.08, 95% CI 1.01–1.15), lower BMI (HR 0.74, 95% CI 0.58–0.96), serum sTREM-1 levels >260 pg/mL (HR 13.93, 95% CI 1.57–58.59), and serum CRP levels >2.6 mg/L (HR 7.85, 95% CI: 1.31–47.05).

**Table 4:** Cox proportional hazard model for on-treatment mortality by clinical characteristics and serum biomarkers levels in patients with pulmonary TB^a^.

	Univariate		Multivariate	
	HR (95% CI)	P value	HR (95% CI)	P value
Age	1.09 (1.02–1.15)	0.006	1.08 (1.01–1.15)	0.019
Male	1.75 (0.38–8.08)	0.467		
BMI	0.68 (0.53–0.87)	0.002	0.74 (0.58–0.96)	0.024
smoking	1.28 (0.43–3.81)	0.663		
Prior TB treatment	1.19 (0.24–6.03)	0.833		
Diabetes	0.87 (0.19–4.01)	0.853		
Renal insufficiency	2.19 (0.28–17.15)	0.454		
COPD	5.01 (0.92–27.25))	0.107		
Malignancy	3.02 (0.86–10.62)	0.101		
sTREM1 >260 pg/mL	8.93 (2.44–32.69)	0.001	13.93 (1.57–58.59)	0.018
sTREM2 >99 pg/mL	5.26 (1.59–17.40)	0.007	0.31 (0.04–2.20)	0.240
CRP >2.6 mg/L	5.85 (1.79–19.14)	0.004	7.85 (1.31–47.05)	0.024

^a^Hazard ratio and 95% confidence interval were derived from the Cox proportional-hazards regression model.

HR, hazard ratio; CI, confidence interval; COPD, chronic obstructive pulmonary disorder; sTREM, soluble triggering receptor expressed on myeloid cells; CRP, C-reactive protein.

### TREM-1 expression in lung tissues

**Figure 4:**
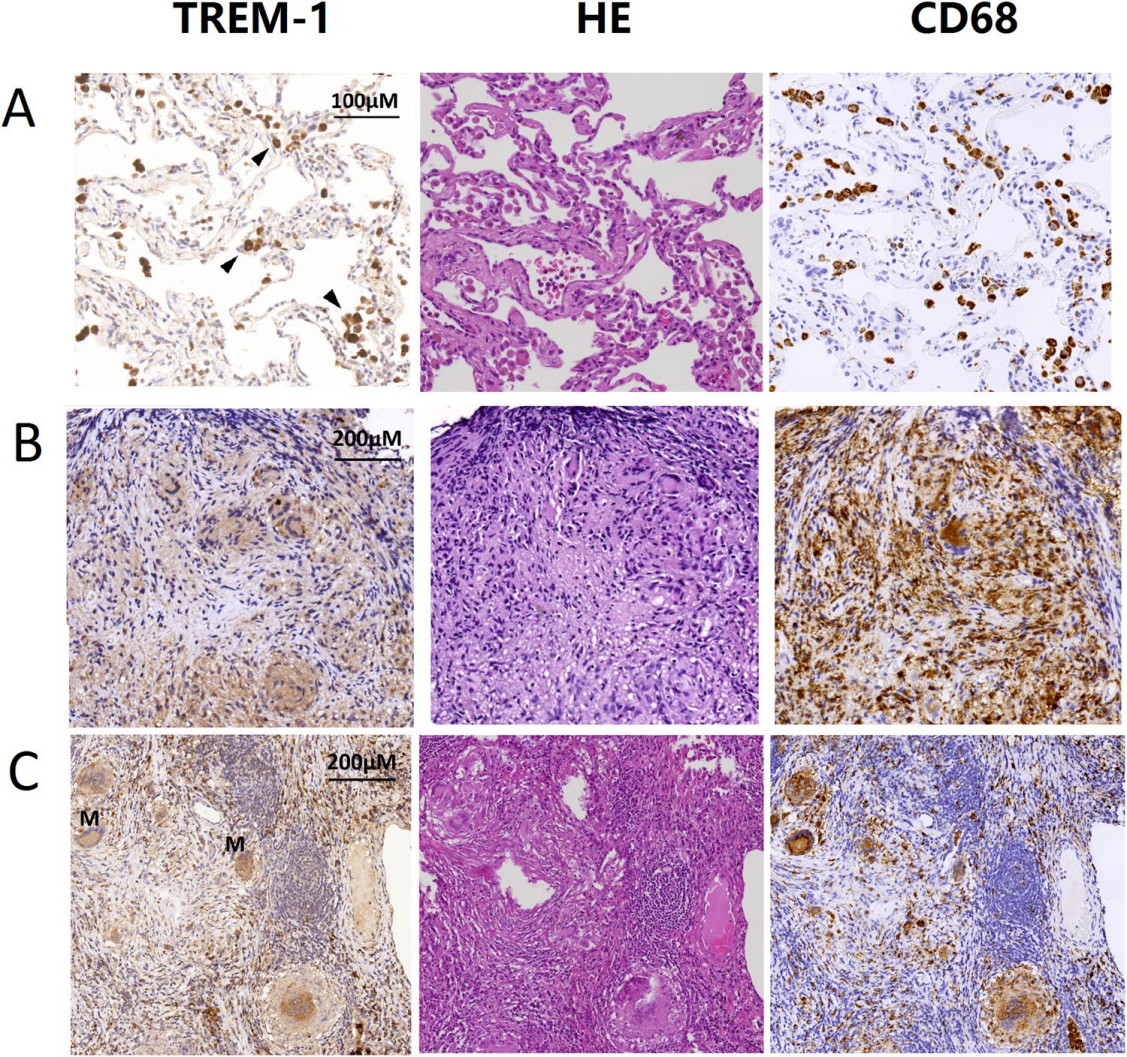
TREM-1-positive cells in lung specimens from patients with pulmonary TB. Immunohistochemistry using an anti-TREM-1 antibody showed that TREM-1-positive cells were localized in the (**A**) alveolar air spaces (*arrow heads*), (**B**,**C**) granulomas and multinuclear giant cells. Immunohistochemistry using an anti-CD68 antibody confirmed that TREM-1-positive cells were mostly macrophages. M indicates multinuclear giant cells. *Arrowheads* indicate TREM-1-positive cells. HE, hematoxylin and eosin stain; TREM, triggering receptor expressed on myeloid cells.

Since the above findings suggested that serum sTREM-1 levels increased in patients with pulmonary TB and significantly correlated with clinical presentations and on-treatment mortality, we further examined TREM-1 expression in lung tissues from two patients with pulmonary TB. Their clinical characteristics and serum biomarkers levels are provided in Supplementary Table 1. As shown in Fig. 4, strong TREM-1 positive cells were identified in the alveolar air spaces. TREM-1-positive cells were also found in granulomas and multinuclear giant cells, although the expression was weaker than that observed in the alveolar airspaces. IHC staining for CD68 confirmed that most TREM-1-expressing cells were human macrophages.

## Discussion

In this prospective observational study, we identified a specific role of sTREM-1 in clinical presentations and treatment response of pulmonary TB. Patients with pulmonary TB had higher serum levels of sTREM-1 as compared to those observed in LTBI cases and non-TB, non-LTBI subjects. Pulmonary TB cases with higher sTREM-1 levels were more likely to have constitutional symptoms, bilateral involvement in chest plain films, and higher MTB burden in sputum. Patients with pulmonary TB with higher sTREM-1 levels also had higher on-treatment mortality. In multivariate analysis, a higher serum level of sTREM-1 was an independent factor associated with on-treatment mortality. We also identified abundant TREM-1-expressing macrophages in lung tissue specimens from pulmonary TB patients. On the contrary, the role of sTREM-2 in clinical manifestations and treatment outcomes of pulmonary TB patients is limited.

TREM-1 is a cell surface protein expressed on myeloid cells and is an important regulator of innate immunity^16^. Through crosstalk between TREM-1 and TLRs, activation of the TREM-1 downstream pathway can amplify the inflammatory responses induced by TLRs such as TLR2 and TLR4^7,17^. Among the various pattern recognition receptors involved in recognizing MTB, TRL2 and TLR4 and their adaptor molecule, MyD88, play central roles in initiating innate immune responses against MTB^18,19^. Interestingly, the early secreted antigen ESAT-6 of MTB can inhibit TLR signaling by directly binding to TLR2, which can lower innate immune responses to infection^20^. Prolonged TLR2 signaling induced by cell wall components of MTB can also induce the production of inhibitory cytokines that limit mitogen-activated protein kinase pathway activation^21^. Considering the immunomodulatory effects of MTB through TLRs, we speculated that TREM-1, owing to its inflammation amplification effects, may act as a supplementary molecule to restore TLR-related immune responses against MTB.

The role of sTREM-1 as a potential biomarker to evaluate disease severity and treatment outcomes has been evaluated in patients with sepsis and pneumonia. Oku *et al*. reported that plasma sTREM-1 levels were significantly higher in patients with sepsis than those in patients with acute respiratory distress syndrome, and plasma sTREM-1 level was positively correlated with severity score and mortality^15^. Another prospective study demonstrated that plasma sTREM-1 level can be used to diagnose sepsis in critically ill patients in combination with other biomarkers^22^. Although a meta-analysis concluded that the performance of sTREM-1 in diagnosing sepsis was only moderate^23^, it suggested that TREM-1 plays a role in sepsis pathogenesis. In line with the findings in sepsis, sTREM-1 is also a useful biomarker for respiratory infections. Wu *et al*. reported that sTREM-1 levels in bronchoalveolar lavage fluid (BALF) significantly increased in patients with ventilator-associated pneumonia, and dynamic changes in sTREM-1 concentration in BALF are prognostically valuable for predicting mortality^24^. Serum levels of sTREM-1 have also been reported to predict bacteremia in patients with community-acquired pneumonia (CAP)^25^. A recent meta-analysis evaluated the prognostic values of sTREM-1 levels in the initial stages of infection and concluded that sTREM-1 alone is insufficient to predict mortality as a biomarker in adult patients with infection^26^. However, elevated sTREM-1 levels were associated with increased risk of death, indicating that TREM-1 is involved in immune responses against infections in humans.

As compared to sepsis and bacterial pneumonia, studies evaluating the role of sTREM-1 in pulmonary TB are relatively limited. Tintinger *et al*. reported that sTREM-1 was detectable in sputum from pulmonary TB patients, but levels did not differ from those observed in patients with CAP^27^. Hung *et al*. analyzed the 6-month mortality in TB patients and demonstrated that serum sTREM-1 level was an independent mortality predictor in multivariate analysis. In the present study, we observed elevated sTREM-1 levels in pulmonary TB patients. We analyzed the clinical characteristics and presentations among pulmonary TB patients with higher and lower sTREM-1 levels, and found that TB patients with higher sTREM-1 levels were older and had trend of more diabetes (data not shown). Furthermore, serum sTREM-1 level was positively correlated with higher bacterial burden in sputum and more advanced lung lesions in chest films. In line with results of previous studies, we found that increased serum sTREM-1 level was an independent predictor for on-treatment mortality. Considering the significant association between increased sTREM1 levels and higher bacterial burden and on-treatment mortality, we speculate that increased sTREM-1 levels in pulmonary TB patients with older age and diabetes may be due to more profound TB diseases in these population. However, the potential causative role of older age and diabetes in escalation of sTREM-1 levels also deserves further evaluation.

Most importantly, we clearly identified TREM-1-expressing macrophages in lung tissues from patients with pulmonary TB. To our knowledge, this is the first study that used IHC staining to demonstrate the presence of TREM-1-expressing macrophages in human lungs infected with MTB. Interestingly, we found that the expression of TREM-1 was stronger in alveolar macrophages than in the macrophages from granulomas. Although the exact mechanism remains unknown, our findings indicate that TREM-1 is highly involved in the initial stages of innate immunity against MTB in the alveolar space, and plays a relatively minor role in the later stages of immune responses, such as granuloma formation. However, our findings should be interpreted with caution. we did not obtain lung tissues from LTBI or non-TB, non-LTBI individuals for comparison in our IHC analysis. The expression of TREM-1 is neither evaluated quantitatively. Therefore, further IHC analysis in lung tissues from non-TB subjects is required to consolidate the potential role of TREM-1 in immune responses against MTB.

It is worth noting that expression of TREM-1 in lung tissue is not specific to pulmonary TB. TREM-1 is also reported to be expressed in tumor-associated macrophages (TAM) in non-small cell lung cancer in previous studies^28,29^. Increased expression of TREM-1 in TAM were reported to predict cancer aggressiveness and associated with worse treatment outcomes. *In vitro* studies demonstrated that the expression of TREM-1 in macrophages in tumor microenvironment is dependent on COX-2 signaling. It suggested that TREM-1 expression in lung tissue is not specific to infectious disease such as pulmonary TB, and is probably also involved in immunomodulation of lung cancer.

Compared to TREM-1, studies evaluating the role of TREM-2 in TB are even scarcer. Hoogendijk *et al*. reported elevated cell-associated TREM-2 expression on monocytes and CD8+ lymphocytes from TB patients, but serum sTREM-2 levels were similar between TB patients and healthy controls^30^. In our study, serum sTREM-2 levels were similar between patients with pulmonary TB, LTBI cases, and non-TB, non-LTBI subjects. Although patients with higher sTREM-2 levels had higher on-treatment mortality, sTREM-2 level was not an independent factor for on-treatment mortality. Unlike TREM-1, the role of TREM-2 in modulating inflammation is much vaguer and further studies are needed to elucidate this issue.

This study had several limitations that are worth noting. As a prospective study, the number of patients with pulmonary TB was relatively small, which may overestimate or underestimate the mortality predictive values of the serum biomarkers. Blood samples for biomarker measurement were collected before initiating anti-TB treatment; therefore, the role of the changing patterns of these biomarkers in treatment outcomes cannot be evaluated. The impact of anti-TB treatment in dynamic changes of these biomarkers is also unknown. Patients with HIV and other comorbidities that may affect immune response were excluded in our study design. Therefore, further studies are needed to validate the mortality-predictive value of sTREM-1 in pulmonary TB patients with compromised immune function. Finally, only patients with pulmonary TB were included in our study for analysis. Therefore, our findings cannot be readily applied to patients with extrapulmonary TB.

In conclusion, this prospective observational study demonstrated that serum levels of sTREM-1 significantly increased in patients with pulmonary TB. Elevated serum levels of sTREM-1 and CRP correlated with more advanced pulmonary involvement, higher bacteria burden in sputum, and were independent factors associated with on-treatment mortality in patients with pulmonary TB. Moreover, for the first time, we demonstrated the high expression of TREM-1 in macrophages from lung tissues of patients with pulmonary TB, although LTBI or non-TB, non-LTBI individuals were not included for IHC analysis. Our findings provided serological and pathological evidence suggesting that TREM-1 is potentially involved in the host immune responses against MTB infection. Further *in vivo* and *in vitro* studies are warranted to explore the exact role of TREM-1 in innate immune responses triggered by MTB infection.

## Methods

### Patients and settings

This was a prospective observational study conducted in a tertiary medical center in northern Taiwan. Newly diagnosed, culture-proven pulmonary tuberculosis patients from August 2014 to July 2016 were eligible for enrollment. We also included individuals of active TB contact for comparison. Active TB patients without pulmonary involvement; individuals younger than 20 years; and patients with active malignancies, human immunodeficiency virus (HIV) infection, autoimmune diseases, and organ transplants were excluded. The demographic characteristics and clinical symptoms/signs were determined based on participant statements obtained in the enrollment interview. The chest plain films of each pulmonary TB patient were reviewed by a pulmonologists and a radiologist, and the radiographic presentations, including extent of lung involvement and the presence of cavitary lesions, were recorded accordingly. This study was approved by the Institutional Review Board of Taipei Veterans General Hospital (IRB number 2014-09-007 A), and written informed consent was obtained from each participant or their authorized representative(s) before enrollment. The methods were carried out in accordance with the approved guidelines.

### Determination of latent TB infection

The Interferon-γ (IFN-γ) release assay (IGRA) was performed in all participants with whole-blood-based QuantiFERON-TB Gold In-Tube (QFT-GIT; Qiagen, Germantown, USA) as previously prescribed^31^. Individuals of active TB contacts were categorized into subgroups of latent TB infection (LTBI) cases and non-TB, non-LTBI subjects based on IGRA results and analyzed accordingly.

### Serum inflammatory biomarker measurement

Peripheral blood samples were collected from the participants on the day of enrollment, which was before the initiation of anti-TB or LTBI prophylactic treatment. We used commercialized enzyme-linked immunosorbent assay kits to measure serum levels of sTREM-1 (Human TREM-1 Quantikine ELISA Kit; R&D Systems, Minneapolis, MN) and sTREM-2 (Human TREM-2/TREM2 ELISA Pair Set; Sino Biological Inc., Beijing, China) in duplicate. Serum C-reactive protein (CRP) levels were measured by the core facility of Taipei Veterans General Hospital using an Abbott Architect C16000 analyzer (Abbott Diagnostics, USA). The measurement of serum biomarkers levels by commercialized ELISA kits were performed in bio-containment level 1 laboratory. Based on the median serum levels of sTREM-1, sTREM2, and CRP, patients with pulmonary TB were categorized into subgroups of high and low serum biomarker levels for further analysis.

### Clinical features and treatment outcomes of pulmonary TB patients

To investigate the role of serum biomarker levels in pulmonary TB disease severity, we analyzed differences in clinical presentations and treatment outcomes of patients with pulmonary TB possessing high and low serum biomarker levels. The clinical presentations we analyzed included constitutional symptoms/signs, respiratory symptoms/signs, radiological presentations, and bacteria burden evaluated by the results of sputum smears. The treatment outcomes evaluated in the present study included two-month smear/culture conversion and on-treatment mortality. Two-month smear/culture conversion was defined as all collected sputum at two months after initiation of anti-TB treatment being smear/culture negative for MTB, and there being no positive cultures thereafter until the completion of anti-TB treatment^32^. On-treatment mortality was defined as patients who died for any reason during the course of anti-TB treatment, which means the observation ended when anti-TB treatment is completed^33^. The treatment duration of the enrolled TB patients ranged from 174 days to 368 days and the median treatment duration was 245 days.

### IHC analysis of TREM-1 expression

To identify TREM-1-expressing inflammatory cells in lung tissues, we obtained lung specimens from two patients with pulmonary TB that were diagnosed based on pathologic characteristics and positive TB cultures of lung specimens. Both their lung specimens showed necrotizing granulomatous inflammation with the presence of mycobacterium, and the in-house TB polymerase chain reaction results were positive. IHC staining for TREM-1 expression analysis was carried out in the core facility of Taipei Veterans General Hospital. In short, 4-μm sections were prepared from formalin-fixed, paraffin-embedded tissues and the IHC staining was performed on a Leica Bond-MAX system (automatic IHC staining system). After deparaffinization, sections were pre-treated using heat-mediated antigen retrieval with sodium citrate buffer (pH 6, epitope retrieval solution 1) for 30 minutes. The section was then incubated with a human anti-TREM-1 antibody (ab93717, Abcam Inc.) at 10 µg/mL (diluted 1/100) for 60 minutes at room temperature, and detected using an HRP-conjugated compact polymer system (Anti-Rabbit IgG–Poly-HRP). The section was blocked with peroxide block (Bond Polymer Refine Detection, Leica Biosystems) for 5 minutes. DAB was used as the chromogen. IHC staining with anti-CD68 antibody (Dako, Glostrup, Denmark) was also performed to identify the presence of human macrophages in lung specimens.

### Statistical analysis

Statistical analysis was carried out using statistical software package (SPSS version 20.0; SPSS Inc., Chicago, IL, USA). Continuous variables between subgroups were compared with independent *t* tests, and categorical variables were compared using Pearson’s chi-square or Fisher’s exact test. Comparisons of serum biomarker levels were carried out using a Mann-Whitney U test. For treatment outcome analysis, patients with pulmonary TB were stratified into subgroups of high and low biomarker levels based on the median serum biomarker level. The optimal cut-off values of serum biomarkers levels for predicting mortality were determined by Receiver Operating Characteristic curves. The Kaplan-Meier method was used to estimate on-treatment survival times of patients with pulmonary TB. A log-rank test was used to compare on-treatment mortality between TB patients with higher and lower serum biomarkers levels. A multivariate Cox proportional hazards regression model with forward stepwise selection procedures was used to identify the risk factors for on-treatment mortality. The parameters included in the model were clinical characteristics, comorbidities, and serum biomarkers levels. A p value less than 0.1 in the univariate analysis was required for a variable to be entered into the multivariate model. All tests were two-tailed, and p values less than 0.05 were considered to be statistically significant.

### Data availability

The datasets generated during and/or analysed during the current study are available from the corresponding author on reasonable request.

## Electronic supplementary material

Supplementary Table 1

## References

[1] Lerner TR (2017). Mycobacterium tuberculosis replicates within necrotic human macrophages. J Cell Biol.

[2] Divangahi M (2009). Mycobacterium tuberculosis evades macrophage defenses by inhibiting plasma membrane repair. Nat Immunol.

[3] Bouchon A, Facchetti F, Weigand MA, Colonna M (2001). TREM-1 amplifies inflammation and is a crucial mediator of septic shock. Nature.

[4] Klesney-Tait J, Turnbull IR, Colonna M (2006). The TREM receptor family and signal integration. Nat Immunol.

[5] McVicar DW (1998). DAP12-mediated signal transduction in natural killer cells. A dominant role for the Syk protein-tyrosine kinase. J Biol Chem.

[6] Arts RJ (2011). TREM-1 interaction with the LPS/TLR4 receptor complex. Eur Cytokine Netw.

[7] Zheng H (2010). MYD88-dependent and -independent activation of TREM-1 via specific TLR ligands. Eur J Immunol.

[8] Bleharski JR (2003). A role for triggering receptor expressed on myeloid cells-1 in host defense during the early-induced and adaptive phases of the immune response. J Immunol.

[9] Haselmayer P, Grosse-Hovest L, von Landenberg P, Schild H, Radsak MP (2007). TREM-1 ligand expression on platelets enhances neutrophil activation. Blood.

[10] Turnbull IR (2006). Cutting edge: TREM-2 attenuates macrophage activation. J Immunol.

[11] N’Diaye EN (2009). TREM-2 (triggering receptor expressed on myeloid cells 2) is a phagocytic receptor for bacteria. J Cell Biol.

[12] Guerreiro R (2013). TREM2 variants in Alzheimer’s disease. N Engl J Med.

[13] Jonsson T (2013). Variant of TREM2 associated with the risk of Alzheimer’s disease. N Engl J Med.

[14] Shi JX (2013). Diagnostic value of sTREM-1 in bronchoalveolar lavage fluid in ICU patients with bacterial lung infections: a bivariate meta-analysis. PLoS One.

[15] Oku R (2013). Differential pattern of cell-surface and soluble TREM-1 between sepsis and SIRS. Cytokine.

[16] Dower K, Ellis DK, Saraf K, Jelinsky SA, Lin LL (2008). Innate immune responses to TREM-1 activation: overlap, divergence, and positive and negative cross-talk with bacterial lipopolysaccharide. J Immunol.

[17] Ornatowska M (2007). Functional genomics of silencing TREM-1 on TLR4 signaling in macrophages. Am J Physiol Lung Cell Mol Physiol.

[18] van de Veerdonk FL (2010). Mycobacterium tuberculosis induces IL-17A responses through TLR4 and dectin-1 and is critically dependent on endogenous IL-1. J Leukoc Biol.

[19] Kleinnijenhuis J, Oosting M, Joosten LA, Netea MG, Van Crevel R (2011). Innate immune recognition of Mycobacterium tuberculosis. Clin Dev Immunol.

[20] Pathak SK (2007). Direct extracellular interaction between the early secreted antigen ESAT-6 of Mycobacterium tuberculosis and TLR2 inhibits TLR signaling in macrophages. Nat Immunol.

[21] Saraav I, Singh S, Sharma S (2014). Outcome of Mycobacterium tuberculosis and Toll-like receptor interaction: immune response or immune evasion?. Immunol Cell Biol.

[22] Gibot S (2012). Combination biomarkers to diagnose sepsis in the critically ill patient. Am J Respir Crit Care Med.

[23] Wu Y (2012). Accuracy of plasma sTREM-1 for sepsis diagnosis in systemic inflammatory patients: a systematic review and meta-analysis. Crit Care.

[24] Wu CL, Lu YT, Kung YC, Lee CH, Peng MJ (2011). Prognostic value of dynamic soluble triggering receptor expressed on myeloid cells in bronchoalveolar lavage fluid of patients with ventilator-associated pneumonia. Respirology.

[25] Ruiz-Gonzalez A (2011). Triggering receptor (TREM-1) expressed on myeloid cells predicts bacteremia better than clinical variables in community-acquired pneumonia. Respirology.

[26] Su L, Liu D, Chai W, Liu D, Long Y (2016). Role of sTREM-1 in predicting mortality of infection: a systematic review and meta-analysis. BMJ Open.

[27] Tintinger GR (2012). Soluble triggering receptor expressed on myeloid cells in sputum of patients with community-acquired pneumonia or pulmonary tuberculosis: a pilot study. Eur J Clin Microbiol Infect Dis.

[28] Ho CC (2008). TREM-1 expression in tumor-associated macrophages and clinical outcome in lung cancer. Am J Respir Crit Care Med.

[29] Yuan Z (2014). TREM-1 is induced in tumor associated macrophages by cyclo-oxygenase pathway in human non-small cell lung cancer. PLoS One.

[30] Hoogendijk AJ (2015). Soluble and cell-associated triggering receptor expressed on myeloid cells-1 and -2 in patients with pulmonary tuberculosis. J Infect.

[31] Feng JY (2013). Characteristics of IFN-gamma responses in IGRA among pulmonary TB suspects in a TB-endemic area. Diagn Microbiol Infect Dis.

[32] Su WJ, Feng JY, Chiu YC, Huang SF, Lee YC (2011). Role of 2-month sputum smears in predicting culture conversion in pulmonary tuberculosis. Eur Respir J.

[33] Feng JY (2011). Initial presentations predict mortality in pulmonary tuberculosis patients–a prospective observational study. PLoS One.

