# Supplementary material for: Role of TREM-1 in pulmonary tuberculosis patients- analysis of serum soluble TREM-1 levels
Source: Sci Rep. 2018 May 29;8:8223. doi: 10.1038/s41598-018-26478-2 (PMC5974358; doi:10.1038/s41598-018-26478-2)
Supplement: Supplementary file 1 — Supplementary Table 1 [file 41598_2018_26478_MOESM1_ESM.docx]

Title:

**Role of TREM-1 in pulmonary tuberculosis patients- analysis of serum soluble TREM-1 levels**

Jia-Yih Feng^1,2,3^, Wei-Juin Su^1, 2^, Sheng-Wei Pan^1,2,4^, Yi-Chen Yeh^5^, Yung-Yang Lin^3, 6, 7,+^, and Nien-Jung Chen^8, +, *^

Running title: Soluble TREM-1 in PTB patients

Jia-Yih Feng, MD

^1^Department of Chest Medicine, Taipei Veterans General Hospital, Taipei, Taiwan

^2^School of Medicine, National Yang-Ming University, Taipei, Taiwan

^3^Institute of Clinical Medicine, School of Medicine, National Yang-Ming University, Taipei, Taiwan

^4^Institute of Public Health, National Yang-Ming University, Taipei, Taiwan

E-mail: swpan25@gmail.com

^5^Department of Pathology and Laboratory Medicine, Taipei Veterans General Hospital, Taipei, Taiwan

^6^Division of Cerebrovascular Diseases, Neurological Institute,

Taipei Veterans General Hospital,

^7^Institute of Brain Science, National Yang-Ming University

^8^Institute of Microbiology and Immunology, School of Life Sciences, National Yang-Ming University

^+^ Yung-Yang Lin and Nien-Jung Chen contributed equally to this work

*Corresponding authors: Nien-Jung Chen Ph.D.

Institute of Microbiology and Immunology, School of Life Sciences, National Yang-Ming University

No.155, Sec.2, Linong Street, Taipei, 112 Taiwan (ROC)

Office number: +886-2-28267106

Fax number: +886-2-28212880

E-mail: [njchen@ym.edu.tw](mailto:njchen@ym.edu.tw)

Supplementary table 1.

Demographic characteristics and serum biomarkers level of the two pulmonary TB patients with IHC analysis in lung tissue^a^

|  | Case 1 | Case 2 |
| --- | --- | --- |
| Mean age | 48 | 54 |
| sex | Male | Male |
| BMI | 22.3 | 25.4 |
| Smoking history | No | Yes |
| BCG vaccination | Yes | Yes |
| Prior TB treatment history | No | No |
| Diabetes | No | Yes |
| Renal insufficiency | No | No |
| COPD | No | No |
| Malignancy | No | No |
| Post gastrectomy | No | No |
| Mean serum biomarker levels |  |  |
| sTREM1 (pg/mL) | 197.5 | 323.5 |
| sTREM2 (pg/mL) | 42.2 | 226 |
| CRP (mg/L) | 1.92 | 0.93 |

BMI, body mass index; BCG, Bacillus Calmette–Guérin; PTB, pulmonary tuberculosis; TB, tuberculosis; LTBI, latent TB infection; COPD, chronic obstructive pulmonary disorder; sTREM, soluble triggering receptor expressed on myeloid cells; CRP, C-reactive protein
